# Uncovering the complex genetics of human temperament

**DOI:** 10.1038/s41380-018-0264-5

**Published:** 2018-10-02

**Authors:** Igor Zwir, Javier Arnedo, Coral Del-Val, Laura Pulkki-Råback, Bettina Konte, Sarah S. Yang, Rocio Romero-Zaliz, Mirka Hintsanen, Kevin M. Cloninger, Danilo Garcia, Dragan M. Svrakic, Sandor Rozsa, Maribel Martinez, Leo-Pekka Lyytikäinen, Ina Giegling, Mika Kähönen, Helena Hernandez-Cuervo, Ilkka Seppälä, Emma Raitoharju, Gabriel A. de Erausquin, Olli Raitakari, Dan Rujescu, Teodor T. Postolache, Joohon Sung, Liisa Keltikangas-Järvinen, Terho Lehtimäki, C. Robert Cloninger

**Affiliations:** 1 Department of Psychiatry, Washington University School of Medicine, St. Louis, MO USA; 2 Department of Computer Science, University of Granada, Granada, Spain; 3 Department of Psychology and Logopedics, University of Helsinki, Helsinki, Finland; 4 Department of Psychiatry, Martin-Luther-University Halle-Wittenberg, Halle, Germany; 5 Department of Epidemiology, School of Public Health, Institute of Health and Environment, Seoul National University, Seoul, Korea; 6 Unit of Psychology, Faculty of Education, University of Oulu, Oulu, Finland; 7 Anthropedia Foundation, St. Louis, MO USA; 8 Department of Psychology, University of Gothenburg, Gothenburg, Sweden; 9 Blekinge Centre of Competence, Blekinge County Council, Karlskrona, Sweden; 10 Fimlab Laboratories, Department of Clinical Chemistry, Faculty of Medicine and Life Sciences, Finnish Cardiovascular Research Center-Tampere, University of Tampere, Tampere, Finland; 11 University Clinic, Ludwig-Maximilian University, Munich, Germany; 12 Department of Clinical Physiology, Faculty of Medicine and Life Sciences, Tampere University Hospital, University of Tampere, Tampere, Finland; 13 Department of Psychiatry and Neurosurgery, University of South Florida, Tampa, FL USA; 14 Department of Psychiatry and Neurology, Institute of Neurosciences, University of Texas Rio-Grande Valley School of Medicine, Harlingen, TX USA; 15 Department of Clinical Physiology and Nuclear Medicine, Turku University Hospital, Turku, Finland; 16 Department of Psychiatry, University of Maryland School of Medicine, Baltimore, MD USA; 17 Rocky Mountain Mental Illness, Research, Education and Clinical Center for Veteran Suicide Prevention, Denver, CO USA; 18 Department of Psychological and Brain Sciences, School of Arts and Sciences, and Department of Genetics, School of Medicine, Washington University School of Medicine, St. Louis, MO USA

**Keywords:** Genetics, Neuroscience, Psychology, Diagnostic markers

## Abstract

Experimental studies of learning suggest that human temperament may depend on the molecular mechanisms for associative conditioning, which are highly conserved in animals. The main genetic pathways for associative conditioning are known in experimental animals, but have not been identified in prior genome-wide association studies (GWAS) of human temperament. We used a data-driven machine learning method for GWAS to uncover the complex genotypic–phenotypic networks and environmental interactions related to human temperament. In a discovery sample of 2149 healthy Finns, we identified sets of single-nucleotide polymorphisms (SNPs) that cluster within particular individuals (i.e., SNP sets) regardless of phenotype. Second, we identified 3 clusters of people with distinct temperament profiles measured by the Temperament and Character Inventory regardless of genotype. Third, we found 51 SNP sets that identified 736 gene loci and were significantly associated with temperament. The identified genes were enriched in pathways activated by associative conditioning in animals, including the ERK, PI3K, and PKC pathways. 74% of the identified genes were unique to a specific temperament profile. Environmental influences measured in childhood and adulthood had small but significant effects. We confirmed the replicability of the 51 Finnish SNP sets in healthy Korean (90%) and German samples (89%), as well as their associations with temperament. The identified SNPs explained nearly all the heritability expected in each sample (37–53%) despite variable cultures and environments. We conclude that human temperament is strongly influenced by more than 700 genes that modulate associative conditioning by molecular processes for synaptic plasticity and long-term memory.

## Introduction

Temperament is classically defined as those aspects of personality that express basic emotions like fear, anger, and disgust, and that are developmentally stable and heritable, rather than learned [1]. However, this classical definition is inadequate because human beings have three major systems of learning and memory with distinctive genetic and biological bases that evolved in succession over the long phylogenetic lineage leading from primitive animals to modern human beings [2–4]. Procedural learning of habits is present in all animals through highly conserved molecular mechanisms of associative conditioning, including classical and operant conditioning [5–9]. In contrast, evidence for intentional cognitive processes, such as purposeful goal-seeking, social reconciliation, and abstract symbolization of facts, are present in the primate lineage of human beings, but not in reptiles [2–4, 10]. Evidence for autonoetic or autobiographical learning appears to be present only with the advent of art and science in modern *Homo sapiens* [2, 11–15].

Early research assessing temperament focused on developmentally stable features of activity and affect, but some recent work has extended assessments of temperament to include aspects of attention and self-regulatory processes that emerged later in evolution and that develop in response to both individual experience and social norms [1, 2, 16]. In contrast, Cloninger took an evolutionary perspective to learning in developing the Temperament and Character Inventory (TCI), defining temperament as that aspect of personality based on associative conditioning [17–19]. The TCI measures four temperament dimensions that have been empirically confirmed by functional brain imaging to quantify individual differences in associative conditioning and related human brain circuitry: Harm Avoidance (i.e., fearful, pessimistic vs. risk-taking, optimistic) [20–22], Novelty Seeking (i.e., impulsive, excitable vs. deliberate, reserved) [23, 24], Reward Dependence (i.e., friendly, sentimental vs. detached, objective) [21, 24], and Persistence (i.e., determined, ambitious vs. easily discouraged, underachieving) [25, 26]. Harm Avoidance is an indicator of negative valence that measures passive avoidance learning and increased sensitivity to fearful stimuli mediated by activation of the amygdala, subgenual cingulate cortex, and the insular salience network [22, 27, 28]. Novelty Seeking is an indicator of positive valence that measures approach to novel stimuli [29, 30], even if they do not predict rewards [24], whereas Reward Dependence is predictive of social affiliation and approach to rewards based on a different pattern of activation of dopaminergic neurons in the nucleus accumbens and substantia nigra [24] and on oxytocinergic neurons in the hypothalamus [31]. Persistence quantifies differences in rates of extinction of intermittently rewarded behaviors in response to frustrative non-reward by activation of a circuit connecting the nucleus accumbens, anterior cingulate, and ventrolateral frontal cortex [25, 26].

Studies of gene expression in response to associative conditioning in experimental animals have consistently documented the activation of specific molecular pathways that trigger synaptic plasticity, which is a fundamental basis for long-term memory [7, 32–34]. The Ras-MEK-ERK cascade (also known as the Mitogen-activated Protein Kinase (MAPK) pathway) and the PI3K-AKT-mTOR cascade are major cellular mechanisms for responding to extracellular stimuli, and their activation triggers intracellular processes that promote synaptic plasticity and associative conditioning, including long-term potentiation (LTP) and long-term depression (LDP) [7, 32, 33, 35]. The cell-surface receptors for these pathways can be activated by a wide variety of somatic, psychological, and social stressors that vary in positive and negative valence and in consequences for survival and reproduction [6, 33, 36]. Changes in these pathways in response to associative conditioning occur in a coordinated manner with related processes including stress reactivity [37], neuronal and glial growth [38], and neurotransmission [39]. Therefore, we hypothesized that genes in the same molecular pathways identified in non-human animals for associative conditioning and related processes would be associated with human temperament profiles. This hypothesis was already supported indirectly by our finding that genes in these pathways were associated with the dependent and apathetic character profiles in which self-regulatory personality traits were inadequate to regulate temperament in a healthy manner, resulting in stress reactivity and ill-health [40].

Unfortunately prior genome-wide association studies (GWAS) of temperament that considered only the average effects of genes have identified few genes associated with personality and have specifically failed to uncover the genes associated with long-term memory whether the TCI or other personality inventories were used [41, 42]. Such failure is an example of the “missing” [43] or “hidden” [44] heritability problem in studies of complex phenotypes. Temperament as measured by the TCI and other inventories is known to be strongly influenced by gene–gene [45–48] and gene–environment interactions [49–51]. Such complexity is expected from the extensive feedback interactions among the molecular pathways that are activated in non-human animals in response to associative conditioning [52].

As in our accompanying GWAS of human character [40], we have chosen to use strictly data-driven methods of deep cluster analysis in GWAS to uncover the complex genotypic and phenotypic architecture of temperament [53–55]. We postulate that the genes in molecular pathways related to temperament are not missing but are distributed in different networks of interacting genes and environments that influence different people [54–57]. More specifically, we hypothesize that the genes associated with temperament will be enriched in the molecular pathways experimentally activated by associative conditioning in non-human animals.

## Subjects and methods

Subjects and methods were the same as detailed in an accompanying paper [40], so essentials are briefly summarized here.

### Description of the samples

Our discovery sample was the Young Finns Study, an epidemiological study of 2149 healthy Finnish children followed regularly from 1980 (ages 3–18 years) to 2012 (ages 35–50 years) [58]. All Finnish subjects (56% women) had thorough standardized genotypic, environmental, and phenotypic assessments, including administration of the TCI [16, 58].

We replicated the results in two independent samples of healthy adults from Germany [59, 60] and Korea [61, 62] in which comparable genotypic and phenotypic features were available (see Supplement). The Korean study involved 1052 unrelated individuals extracted from a national register (aged 28–81 years, 57% women). The German study involved 902 subjects (aged 20–74 years, 49% women) randomly selected from the Munich city register and screened to exclude anyone with a history of psychiatric illness in themselves or their first-degree relatives.

### Personality assessment

All subjects completed the TCI to assess seven heritable dimensions of personality [18, 63]. The TCI measures four well-validated dimensions of temperament (Novelty Seeking, Harm Avoidance, Reward Dependence, Persistence) and three dimensions of character, as described in the “Introduction” and in more detail in Supplementary Section 1 and Table S1 [18, 63]. The 12 temperament subscales from the TCI were used as the primary phenotypic data in all three samples (Supplementary Section 2 and Table S1).

### Personality health indices

People at risk of unhealthy personality were identified as the bottom decile of the sum of TCI Self-directedness and Cooperativeness [64], a previously validated indicator of ill-being [65, 66]. In contrast, people with healthy personalities were identified as the top decile of the product of all three TCI character traits, a previously validated indicator of well-being [64, 67, 68]. Our ill-being and well-being indices were used to measure the health status of subjects consistently in all three samples.

We also identified an empirical index of temperament (Supplementary Section 3 and Table S2) as a single comprehensive measure of temperament that could be used in SNP-set Kernel Association Test (SKAT) [56, 57] and heritability analyses.

### Genotyping

The Finnish sample was genotyped by using Illumina Human670-Quad Custom, (i.e., Illumina 670k custom) arrays [69]. The Korean sample used Affymetrix Genome-Wide Human SNP Array 6.0 and Illumina HumanCore [61]. The German sample used Affymetrix Genome-Wide Human SNP Array 6.0, Illumina OMNI Express and the 300 Array, pre-phased and imputed with SHAPEIT2 and IMPUTE2. Some German individuals had also been genotyped on Illumina Omni1-Quad. Quality control was performed for all samples as in prior work [55] (Supplementary Section 3).

After quality control, the PLINK software suite [70] was used to reduce the large search space by pre-selecting a subset of SNPs using a generously inclusive threshold (*p*-value < 0.01 without Bonferroni correction) for possible association with temperament, taking gender and ethnicity into account as covariates of the individual SNPs, as detailed in an accompanying paper [40]. We accounted for ethnicity in each sample by using the first three principal components for ancestral stratification of SNP genotypes (Supplementary Section 3) [71].

### Computational procedures

The cluster analyses used the Generalized Factorization Method [72–75] including Non-negative Matrix Factorization (NMF), which optimizes pattern recognition and naturally occurring associations between patterns across different types of data. The clustering was entirely data-driven without restrictive assumptions about the number or content of the clusters [54], as detailed elsewhere [53–55, 72, 76]. The steps of this analytic procedure are summarized and schematically related to unsupervised Deep NMF Learning in Supplementary Figure S1. The advantages of this clustering approach over alternative analyses of single or multiple markers are described in Supplementary Section 4.

Our web server application for Phenotype–Genotype Many-to-many Relations Analysis (PGMRA) in GWAS is published [54] and available online at http://phop.ugr.es/fenogeno. The PGMRA method and algorithm are also summarized in Supplementary Sections 5 and 6, which include a semi-supervised classifier of phenotypes from genotypes. PGMRA properly accounts for Linkage Disequilibrium (LD) efficiently (i.e., without loss of information about complex genotypic–phenotypic relations) (Supplementary Section 4). Statistical analysis correcting for multiple comparisons, as well as gender and ethnicity as covariates of the SNP sets, was performed by SKAT [56, 57], also accessible via PGMRA. Heritability was estimated from a trimmed regression of SNPs on the empirical index of temperament controlling for outliers and environmental variables [77, 78] (see also Supplementary Section 7).

Replicability of results was evaluated in the three independent samples for SNP sets, phenotypic sets, and genotypic–phenotypic relations using multi-objective optimization techniques [55], as detailed in Supplementary Section 8. The PGMRA classifier was used to predict temperament phenotypes from the genotypic sets (Supplementary Section 9). Further details are available in Supplementary Information and elsewhere [72–75].

## Results

### Identifying SNP sets as candidates for causal variability

**Fig. 1:**
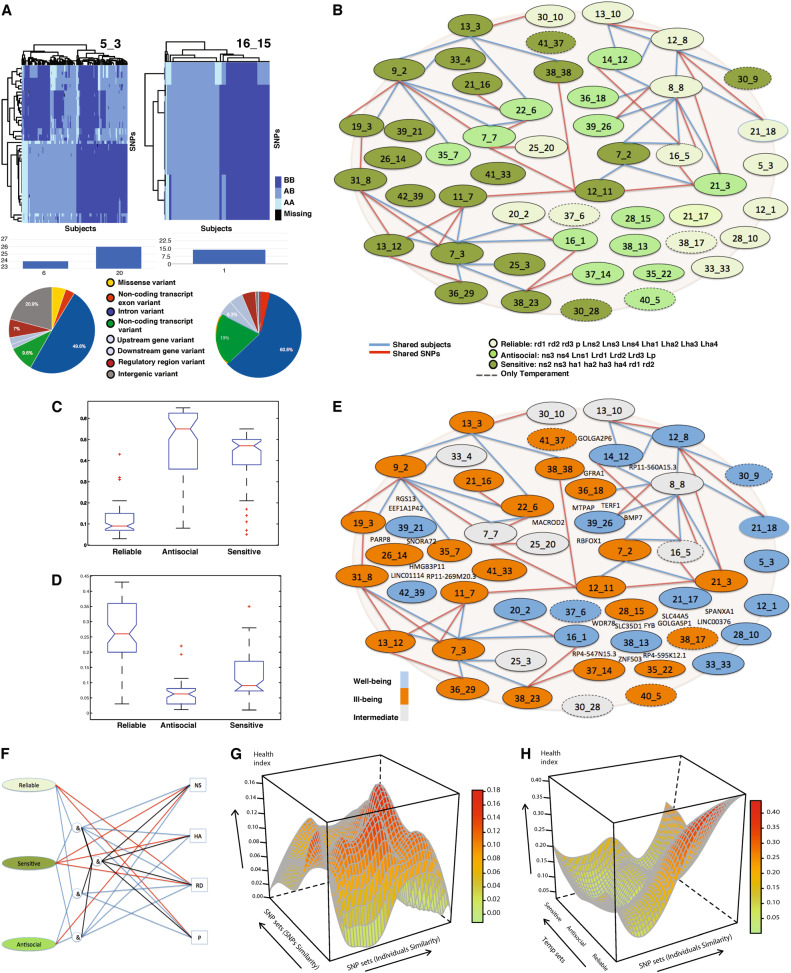
**a** Two examples of SNP sets are represented as Heat Map submatrices or biclusters. SNP sets were identified by distinct patterns of molecular features of SNPs in subgroups of subjects. Allele values are indicated as BB (dark blue), AB (intermediate blue), AA (light blue), and missing (black). SNP sets were labeled for specificity by a pair of numbers representing the maximum number of clusters from which the bicluster was selected (e.g., 16 clusters may produce more specific than 5) and the order in which they were selected by the method (e.g., 3rd bicluster or factor selected by FNMF when the maximum number of clusters was 5) and usually have a prefix G for genotype or P for phenotype. Only a subset of optimal and cohesive sets are selected across all number of clusters (see Supplementary Methods). The SNPs within each SNP set can map to different chromosomes (e.g., 6 and 20) and exhibit distinct molecular consequences (see Supplementary Table S3). The pie chart shows the percentage of SNPs within a SNP set that belong to each type of consequence. **b** Dissection of a GWAS in a Finnish population to identify the genotypic and phenotypic architecture of personality measured by the TCI. The genotypic network is depicted as nodes (SNP sets) linked by shared SNPs (blue lines) and/or subjects (red lines). Each SNP set maps to one or more genes (see Supplementary Table S6 for a full list of genes associated with each SNP set). SNP sets associated with each of the three general temperament profiles are distinguished by color-coding as shown in the legend (see Table 3). **c**, **d** Comparison of level of ill-being (**c** where high values indicate ill-being) and for level of well-being (**d** where high values indicate well-being) in groups of subjects with each of the three temperament profiles specified by both phenotypic and genotypic information (evaluated by ANOVA). (Compare with either genetic or phenotypic assessment alone in Supplementary Figure S5.) **e** Variation in health status of SNP sets: well (blue, see (**d**)), ill (orange, see (**c**)), intermediate (gray). **f** 19 genotypic–phenotypic pipelines connect different sets of genes to the same temperament dimension (see also Supplementary Tables S9–S11). Red lines indicate direct connections, whereas blue lines and “&” indicate composite connections. **g** Surface showing the pattern of health status of the subjects in this study based on SNP set information only (i.e., interpolation from Table 1). The probability of well-being in the *z*-axis varies from high (red for high well-being) to low (green). The order of the SNP sets is based on shared subjects (*x*-axis) and on shared SNPs (*y*-axis) measured by hypergeometric statistics, so SNP sets sharing more SNPs and/or subjects are nearby. (See ill health surface in Supplementary Figure S3.) **h** Surface showing the pattern of health status of subjects based on both genotypic information (SNP sets) and phenotypic information (temperament sets) (as in Table 3). The probability of well-being in the *z*-axis varies from high (red, high well-being) to low (green). The sharing of subjects is shown for both SNP sets (*x*-axis) and temperament sets (*y*-axis). (See ill health surface in Supplementary Figure S4.)

**Fig. 2:**
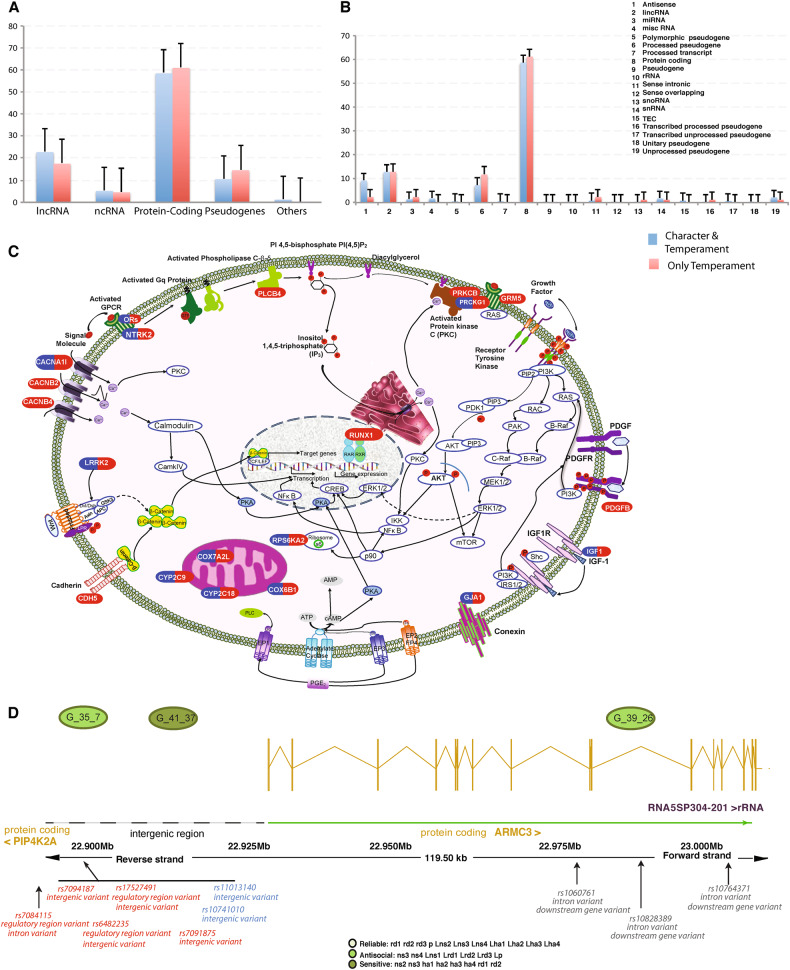
**a**, **b** Types of genetic variants mapped by SNP sets associated with temperament. **a** Specific molecular consequences (Supplementary Table S5) and **b** their subtypes. Genes related only to temperament sets (red) were less often protein coding and more often RNA genes than those also associated with temperament sets (blue color). **c** Cell displaying the molecular pathways containing genes associated with the Sensitive and Antisocial profiles. The uncovered genes influence the Ras-MEK-ERK (MAPK), PI3K-AKT-mTOR, and Protein Kinase A, B, C pathways that regulate associative conditioning (see also Supplementary Tables S4, S7). **d** Multiple SNPs within a SNP set can affect a single or multiple genes in many ways (Supplementary Table S3). The PIP4K2A, the ARMC3 divergent regulatory region, and the ARMC3 coding region are illustrated. SNPs in the SNP set G_41_37 may affect regulatory regions (thereby inhibiting transcription), whereas SNPs from SNP set 39_26 are mostly located in intronic regions (thereby blocking or decreasing protein production). The SNP sets are associated with profiles exhibiting distinct temperament features (sensitive vs. antisocial)

902 Non-identical but possibly overlapping SNP sets were exhaustively identified by PGMRA in the Finnish sample without knowledge of the phenotype, as in our analysis of character [40]. Among these, the SNP sets related to temperament had different numbers of subjects and/or SNPs and associated health risks (Table 1, Supplementary Table S2). The SNPs mapped to diverse classes of genetic variants dispersed across all the chromosomes (Figs. 1a and 2a; Supplementary Figure S2, Supplementary Table S3).

**Table 1:** Description of 51 SNP sets associated with Temperament sets (*p* < 1E−05)

Finnish sample										Probability of health		# Gs
SNP sets	SNP set names	Character	% Coding	SKAT *p*-value	Average SNPs	Best SNP	Worst SNP	# Subjects	# SNPs	Well- being	Ill-being	
G_13_3	ERK-conditioned impulsivity		71	7.38E−14	1.50E−01	1.46E−04	1.00E+00	95	158	0.03	0.32	21
G_8_8	Global inositol/chemokine pathways	Organized	60	1.06E−14	4.39E−01	5.04E−05	1.00E+00	224	611	0.08	0.07	286
G_13_10	Cholinergic neuromodulation		71	3.13E−08	9.38E−02	8.81E−06	5.99E−01	148	57	0.09	0.08	17
G_21_18	Cognitive flexibility		73	2.75E−05	3.53E−01	1.10E−03	1.00E+00	116	47	0.13	0.09	15
G_30_10	TNF-based resilience		50	1.21E−05	2.33E−01	1.74E−04	1.00E+00	47	45	0.09	0.06	6
G_7_3	Neurogenesis	Organized	66	3.32E−06	4.07E−01	4.02E−05	1.00E+00	133	364	0.17	0.36	128
G_38_23	Sensory sensitivity		63	7.60E−07	1.23E−02	3.90E−04	1.12E−01	39	37	0.05	0.49	16
G_25_3	Acetylcholine biosynthesis		50	2.06E−06	3.62E−03	1.66E−04	1.54E−02	16	31	0.13	0.50	2
G_31_8	Neurotrophin	Organized	57	4.34E−16	2.55E−01	4.02E−05	1.00E+00	54	183	0.09	0.54	60
G_28_15	Estrogen neuroplasticity	Dependent	52	3.10E−06	3.66E−01	2.05E−04	1.00E+00	101	123	0.08	0.38	29
G_11_7	HPA stress reactivity		64	9.17E−10	1.49E−01	1.66E−04	1.00E+00	26	92	0.08	0.38	11
G_26_14	Glucose transport	Apathetic	60	1.12E−07	2.78E−01	1.31E−04	1.00E+00	46	75	0.09	0.24	25
G_41_33	GPCR neuroplasticity	Dependent	47	1.47E−06	2.70E−01	7.41E−04	1.00E+00	56	76	0.11	0.21	15
G_21_16	Acetylcholine biosynthesis		100	8.24E−06	2.41E−03	1.66E−04	1.54E−02	37	26	0.14	0.22	1
G_38_38	Ion permeability		67	2.67E−12	1.60E−01	1.08E−04	1.00E+00	38	79	0.00	0.16	18
G_7_2	GPCR dysregulation	Dependent	61	2.12E−18	3.39E−01	2.95E−04	1.00E+00	211	303	0.09	0.23	147
G_12_11	Ras-Akt interaction		75	8.00E−08	5.89E−02	2.77E−04	5.95E−01	105	44	0.03	0.21	4
G_16_1	PI3K-based memory		64	1.72E−05	2.61E−01	3.35E−04	1.00E+00	108	53	0.16	0.27	11
G_37_14	Neuroexcitability		58	1.25E−05	2.50E−02	1.74E−04	8.12E−01	21	42	0.05	0.14	12
G_13_12	Acetylcholine biosynthesis		100	6.26E−06	8.60E−02	1.66E−04	1.00E+00	78	47	0.23	0.31	1
G_12_1	Episodic learning	Creative	61	2.92E−13	2.44E−01	1.08E−04	1.00E+00	146	189	0.20	0.06	64
G_28_10	WD/CDK neuroplasticity		50	7.40E−06	3.11E−02	3.22E−04	2.76E−01	46	30	0.15	0.15	8
G_5_3	Regulation pathways		0	1.40E−05	4.24E−03	1.74E−04	3.74E−02	172	50	0.28	0.05	2
G_38_13	Glucuronidase habit extinction		57	6.25E−06	6.73E−02	1.74E−04	7.31E−01	60	63	0.13	0.20	7
G_35_22	PI3K-based memory		80	2.90E−07	1.04E−01	1.31E−04	5.71E−01	43	36	0.07	0.21	5
G_33_4	ERK-PKA interaction		50	4.53E−05	1.80E−01	3.35E−04	1.00E+00	24	51	0.00	0.33	6
G_12_8	Neuroprotection	Resourceful	63	3.30E−22	2.68E−01	6.89E−05	1.00E+00	173	285	0.09	0.03	111
G_39_21	RGS negative emotionality		60	8.48E−06	1.03E−01	3.22E−04	7.53E−01	56	37	0.11	0.07	5
G_7_7	Olfaction	Dependent	52	9.84E−08	3.41E−01	1.66E−04	1.00E+00	145	193	0.03	0.10	58
G_21_3	Cellular senescence	Apathetic	64	8.23E−07	3.00E−01	1.52E−04	1.00E+00	60	117	0.10	0.23	39
G_39_26	mTOR myelination		62	1.08E−09	2.89E−01	5.50E−05	1.00E+00	20	118	0.20	0.30	26
G_42_39	Approach-avoidance conflict		45	2.42E−06	2.28E−01	1.31E−04	1.00E+00	19	52	0.16	0.11	11
G_35_7	PI3K-based memory		67	2.64E−05	2.15E−01	6.19E−04	9.15E−01	32	35	0.09	0.13	12
G_19_3	Glucuronidase habit extinction		80	1.05E−05	6.97E−02	3.35E−04	1.00E+00	48	61	0.08	0.17	5
G_22_6	Blood-brain barrier	Dependent	60	1.09E−07	2.67E−01	1.66E−04	1.00E+00	37	93	0.08	0.16	30
G_20_2	Enhanced memory	Creative	78	2.39E−07	3.15E−01	1.08E−04	1.00E+00	25	80	0.24	0.12	18
G_21_17	TGFβ resistance to aging		65	2.38E−05	3.38E−01	4.23E−04	1.00E+00	67	105	0.18	0.09	26
G_36_18	Brain-RNA-biogenesis		0	1.50E−06	2.06E−03	2.05E−04	8.20E−03	19	25	0.05	0.26	4
G_36_29	Electron transport	Organized	57	4.26E−08	3.57E−01	6.25E−05	1.00E+00	25	185	0.08	0.48	49
G_14_12	Ras-based stress memory		55	1.84E−07	2.58E−01	8.81E−06	1.00E+00	83	74	0.12	0.07	22
G_25_20	Fatty acid oxidation		67	9.84E−07	1.03E−01	1.66E−04	7.16E−01	33	62	0.03	0.12	3
G_33_33	TGFβ memory enhancement		62	1.36E−07	1.65E−01	1.31E−04	1.00E+00	49	47	0.10	0.08	13
G_9_2	Serotonin–cytokine interaction		73	1.33E−05	3.16E−02	3.35E−04	1.00E+00	140	56	0.04	0.24	11
G_30_9	Erk-IP3-PKC Interaction		75	2.80E−16	2.00E−01	1.80E−05	1.00E+00	69	138	0.17	0.14	52*
G_38_17	MAPK memory enhancement		46	8.72E−06	2.20E−01	5.60E−04	1.00E+00	14	42	0.00	0.43	13*
G_40_5	Mannosidase habit extinction		67	3.40E−05	5.49E−02	3.22E−04	2.76E−01	16	30	0.06	0.38	3*
G_30_28	Hippocampal synaptic plasticity		30	2.64E−06	2.76E−01	1.31E−04	1.00E+00	34	53	0.06	0.12	10*
G_16_5	Erk-IP3-PKC interaction-based stress memory		71	6.39E−10	3.87E−01	4.62E−04	1.00E+00	87	324	0.09	0.15	1*
G_16_15	IL-2 neuroimmune response		43	3.E−04	9.20E−01	4.94E−01	1.00E+00	94	14	0.16	0.15	7**
G_37_6	Methylation-based gene silencing		41	5.00E−08	1.13E−01	7.93E−04	1.61E−01	26	34	0.35	0.25	23^#^
G_41_37	PI3K-MAPK cognitive function		51	2.08E−05	4.95E−02	8.22E−04	2.96E−01	41	38	0.10	0.25	11^#^

The SNP sets are named based on molecular pathways and neuronal functions of the genes that distinguish the sets from one another (see Supplementary Table S4). % coding indicates the percentage of protein-coding genes. Strengths of association are compared for the SNP set, the best SNP, and average SNP based on SKAT *p*-values. The number of subjects and SNPs comprising each SNP set is specified. The probabilities of the well-being and ill-being are given for subjects in each SNP set (see also Supplementary Table S2). Character indicates the association of the set with the Character phenotype (published elsewhere [40]). # Gs indicates the number of genes mapped by the SNP sets (Figure S6), where genes can be mapped by more than one SNP set

*indicates SNP-sets directly associated only with temperament sets

### Identifying clusters of subjects with distinct temperament profiles

118 Temperament sets were exhaustively identified by PGMRA in the Finnish sample using the 12 temperament subscales without knowledge of the genotype. These fine-grained sets were identified in clustering solutions with the possible number of sets ranging from 2 to 15. Hierarchically clustering these 118 fine-grained sets with PGMRA, we identified 3 temperament super-sets that minimized the Cophenetic Correlation Coefficient (Table 2). In other words, 3 groups of people had highly distinct temperament profiles.

**Table 2:** Description of the 5 Character Profiles (supersets) and composite character sets identified by PGMRA from profiles of TCI subscales (Y=Yes)

Char sets	Supersets	Name	sd1	sd2	sd3	sd4	sd5	co1	co2	co3	co4	co5	st1	st2	st3	Lsd1	Lsd2	Lsd3	Lsd4	Lsd5	Lco1	Lco2	Lco3	Lco4	Lco5	Lst1	Lst2	Lst3	#S	Well Being	Ill Being
C_14_8	1	Resourceful			Y	Y																						Y	79	0.01	0.01
C_10_7	1		Y		Y	Y					Y																		102	0.41	0
C_10_6	1		Y		Y														Y					Y					33	0.03	0.38
C_14_13	2	Organized					Y	Y	Y	Y	Y															Y	Y		92	0.09	0
C_14_9	2						Y	Y	Y		Y	Y	Y			Y											Y		42	0.02	0.29
C_9_8	2			Y	Y	Y	Y	Y	Y	Y	Y			Y											Y	Y		Y	72	0.12	0
C_12_9	2		Y	Y	Y		Y	Y		Y	Y								Y										41	0.54	0
C_6_5	2		Y	Y	Y	Y	Y	Y	Y	Y	Y	Y														Y	Y		56	0.07	0
C_8_7	2		Y	Y	Y	Y	Y	Y	Y	Y	Y	Y														Y	Y	Y	161	0.04	0
C_5_1	2		Y	Y	Y	Y	Y	Y	Y	Y	Y	Y														Y	Y	Y	169	0.05	0
C_3_1	2		Y	Y	Y	Y	Y	Y	Y	Y	Y	Y														Y	Y	Y	293	0	0
C_4_4	2		Y	Y	Y	Y	Y	Y	Y	Y	Y	Y														Y	Y	Y	190	0.01	0
C_7_7	2		Y	Y	Y		Y	Y	Y	Y	Y	Y														Y	Y	Y	101	0.07	0
C_9_6	2		Y	Y	Y		Y	Y	Y	Y	Y	Y							Y							Y	Y	Y	61	0.11	0.02
C_7_5	2					Y		Y	Y	Y																		Y	34	0.5	0.06
C_9_1	2		Y	Y	Y	Y		Y	Y	Y														Y		Y			46	0	0.02
C_15_5	3	Creative									Y				Y														100	0.72	0.03
C_12_7	3												Y		Y														52	0.9	0.04
C_11_3	3												Y	Y	Y														34	0.97	0.03
C_13_1	3				Y									Y	Y														42	0.9	0.02
C_14_1	3		Y	Y								Y	Y		Y				Y		Y						Y		28	0.25	0.11
C_7_2	3		Y	Y	Y		Y				Y	Y	Y	Y	Y														66	0.98	0
C_8_8	3		Y	Y	Y		Y	Y	Y	Y	Y		Y	Y	Y														39	1	0
C_4_3	3		Y	Y	Y		Y	Y	Y	Y		Y	Y	Y	Y														72	0.97	0
C_5_5	3			Y	Y		Y	Y	Y	Y	Y	Y	Y	Y	Y														73	1	0
C_6_1	3			Y	Y	Y		Y	Y	Y	Y	Y	Y	Y	Y														32	1	0
C_3_3	3		Y	Y	Y	Y	Y	Y	Y	Y	Y	Y	Y	Y	Y														135	0.92	0
C_9_2	3												Y	Y	Y	Y			Y					Y					4	0.05	0.2
C_15_7	4	Dependent									Y																	Y	121	0.03	0.02
C_14_5	4					Y												Y										Y	55	0	0.29
C_15_13	4															Y	Y	Y											29	0	0.79
C_12_6	4					Y					Y					Y	Y	Y		Y									44	0.09	0.41
C_4_2	4					Y					Y	Y				Y	Y	Y		Y									40	0	0.45
C_7_4	4										Y	Y					Y	Y				Y				Y	Y		23	0	0.74
C_5_3	4					Y					Y	Y			Y		Y	Y		Y						Y	Y		48	0.02	0.29
C_6_3	4					Y					Y	Y					Y	Y		Y						Y	Y	Y	37	0	0.32
C_9_5	4					Y		Y	Y	Y	Y	Y			Y		Y	Y								Y	Y		31	0	0.26
C_10_2	5	Apathetic																								Y	Y	Y	116	0.01	0
C_11_4	5																									Y	Y	Y	50	0	0.28
C_8_3	5																					Y				Y	Y	Y	39	0	0.64
C_14_11	5																				Y	Y	Y	Y		Y	Y		62	0	0.47
C_10_8	5																Y			Y	Y	Y	Y		Y	Y	Y	Y	33	0	0.79
C_3_2	5															Y	Y	Y	Y	Y	Y	Y	Y	Y	Y				53	0	1
C_12_5	5									Y	Y	Y						Y								Y	Y	Y	22	0	0.09
C_14_7	5														Y	Y	Y	Y	Y										4	0	1
C_13_3	5		Y				Y	Y											Y					Y					4	0	0.25
C_14_3	5																							Y					32	0.06	0.38
C_11_10	5					Y		Y						Y			Y	Y		Y				Y	Y				38	0.03	0.5
C_15_1	5														Y						Y			Y					21	0.14	0.52
C_7_3	5															Y			Y										15	0	1
C_8_6	5															Y		Y	Y										7	0	1
C_9_3	5															Y	Y	Y	Y		Y	Y	Y	Y	Y		Y	Y	7	0	1
C_15_15	5			Y	Y											Y													33	0	0.3
C_11_6	5																		Y					Y					28	0	0.71
C_12_4	5				Y												Y		Y	Y	Y	Y							34	0.03	0.53
**Consensus sets**			**sd1**	**sd2**	**sd3**	**sd4**	**sd5**	**co1**	**co2**	**co3**	**co4**	**co5**	**st1**	**st2**	**st3**	**Lsd1**	**Lsd2**	**Lsd3**	**Lsd4**	**Lsd5**	**Lco1**	**Lco2**	**Lco3**	**Lco4**	**Lco5**	**Lst1**	**Lst2**	**Lst3**			
Resourceful			Y		Y	Y																									
Organized			Y	Y	Y	Y	Y	Y	Y	Y	Y	Y														Y	Y	Y			
Creative			Y	Y	Y	Y	Y	Y	Y	Y	Y	Y	Y	Y	Y																
Dependent						Y					Y	Y				Y	Y	Y		Y						Y	Y				
Apathetic																Y	Y	Y	Y	Y	Y	Y	Y	Y	Y	Y	Y	Y			

[TCI subscales are indicated Self-directedness (sd1 to sd5), Cooperativeness (co1 to co5), and Self-transcendence (st1 to st3). Subscale values were divided by median split into high and low scores (distinguished by L before the low scores). The number of subjects in each character set is specified (#S). The probabilities of well-being and of ill-being are shown for subjects in each character set (see also Supplementary Table S2)]

The three temperament profiles were named Reliable, Antisocial, and Sensitive based on traditional labels for their prominent features [17]. People in the Reliable profile were high in Reward Dependence (i.e., sentimental, friendly, approval-seeking), high in Persistence (i.e., determined), low in Novelty Seeking (i.e., deliberate, thrifty, orderly), and low in Harm Avoidance (i.e., optimistic, confident, outgoing, vigorous). This profile frequently is associated with healthy and trustworthy behavior (Table 2). In contrast, people in the Antisocial profile were low in Reward Dependence (i.e., cold, detached, independent), low in Persistence (i.e., easily discouraged), and high in Novelty Seeking (i.e., extravagant, rule-breaking, but not inquisitive), which is frequently associated with unhealthy antisocial conduct (Table 2). People with the Sensitive profile were high in Harm Avoidance (i.e., pessimistic, fearful, shy, and fatigable), high in Novelty Seeking (i.e., impulsive, extravagant), and high in Reward Dependence (i.e., sentimental, friendly), which is frequently associated with approach-avoidance conflicts and emotional sensitivity (Table 2).

### Prediction of temperament profiles by SNP sets

We computed the association of SNP sets with temperament in Finnish subjects. SKAT showed that the association of the empirical index of temperament with particular SNP sets was stronger than with the average effects of their constituent SNPs (Table 1). We found 51 SNP sets had significant associations with temperament (*p* < 4E−04). SNP sets were labeled by a genotypic identification “G”, followed by 2 numbers indicating the maximum number of clusters and the order of their selection by the algorithm. For example, the SNP set G_13_3 has a *p*-value of 7.38E−14, whereas the best and average SNPs within this set have 1.46E−04 and 1.50E−01 *p*-values, respectively (Table 1). SKAT [56] and PLINK [70] methods estimated similar *p*-values for the individual SNPs (*R*^2^ = 0.95, *F* statistics, *p* < 1E−41), which showed that SKAT did not inflate results.

The 51 SNP sets associated with temperament are described in Table 1. We assigned names to the SNP sets based on prominent molecular processes and pathways that distinguished them (Supplementary Table S4). The temperament-related SNP sets were comprised of networks of SNPs that mapped to 736 genes, nearly all of which are known to influence individual differences in brain functions. In particular, these SNP sets were involved in the regulation of synaptic plasticity, long-term memory based on associative conditioning (long-term potentiation and depression, fear conditioning, reward reinforcement, habit extinction), and related processes involving stress reactivity, neurotransmission (cholinergic, monoaminergic, GABAergic, glutaminergic), resistance to aging, neuronal and glial growth, myelination, and energy production (Table 1, Supplementary Tables S4–S6).

### Complex genotypic–phenotypic relationships in temperament profiles

We found 44 of the 118 temperament sets were significantly associated with particular SNP sets (Hypergeometric statistics, 1E−11 < *p* < 1E−03, Table 3). The genotypic–phenotypic relations were complex, demonstrating pleiotropy and heterogeneity. For example, G_13_3 (ERK-conditioned impulsivity) is comprised of multiple genes that regulate behavioral disinhibition in associative learning tasks, such as DAB1 and CDH13 (Table 1, Supplementary Table S4); it was frequently associated with sensitive temperament sets, but sometimes with antisocial or reliable profiles (Table 3). The 44 temperament sets were associated with the 51 SNP sets in 158 relationships that were significant by a permutation test (Table 3, empirical *p* < 4.6E−03).

**Table 3:** The strength of the genotypic–phenotypic relationships among SNP and Temperament sets, and their corresponding health measurements

Temp sets^i^	Temperament supersets	SNP sets	Hypergeometric T-G	Health risk					
				Temp set		SNP sets		Relationships	
				Well-being	Ill-being	Well-being	Ill-being	Well-being	Ill-being
T_10_1	Antisocial	G_12_11	1.16E−04	0.02	0.12	0.03	0.21	0.03	0.21
T_10_1	Antisocial	G_13_3	7.01E−06	0.02	0.12	0.03	0.32	0.03	0.32
T_10_5	Reliable	G_13_10	4.87E−03	0.32	0.00	0.09	0.08	0.32	0.08
T_10_5	Reliable	G_21_18	3.75E−07	0.32	0.00	0.13	0.09	0.32	0.09
T_10_5	Reliable	G_8_8	2.24E−04	0.32	0.00	0.08	0.07	0.32	0.07
T_11_1	Sensitive	G_30_9	3.32E−03	0.28	0.05	0.17	0.14	0.28	0.14
T_11_11	Reliable	G_30_10	1.71E−03	0.08	0.02	0.09	0.06	0.09	0.06
T_11_7	Sensitive	G_11_7	4.47E−03	0.04	0.47	0.08	0.38	0.08	0.47
T_11_7/T_4_3^i^	Sensitive	G_13_3	2.04E−08	0.04	0.47	0.03	0.32	0.04	0.47
T_11_7	Sensitive	G_21_16	2.62E−03	0.04	0.47	0.14	0.22	0.14	0.47
T_11_7/T_4_3^i^	Sensitive	G_21_16	2.88E−06	0.04	0.47	0.14	0.22	0.14	0.47
T_11_7	Sensitive	G_26_14	1.62E−04	0.04	0.47	0.09	0.24	0.09	0.47
T_11_7/T_4_3^i^	Sensitive	G_28_15	6.87E−06	0.04	0.47	0.08	0.38	0.08	0.47
T_11_7/T_4_3^i^	Sensitive	G_31_8	4.77E−06	0.04	0.47	0.09	0.54	0.09	0.54
T_11_7	Sensitive	G_38_38	4.04E−04	0.04	0.47	0.00	0.16	0.04	0.47
T_11_7	Sensitive	G_41_33	3.27E−03	0.04	0.47	0.11	0.21	0.11	0.47
T_11_7	Sensitive	G_7_3	2.25E−03	0.04	0.47	0.17	0.36	0.17	0.47
T_11_7/T_4_3^i^	Sensitive	G_9_2	7.69E−08	0.04	0.47	0.04	0.24	0.04	0.47
T_12_4	Reliable	G_12_11	4.28E−03	0.03	0.11	0.03	0.21	0.03	0.21
T_12_4	Reliable	G_13_3	3.03E−05	0.03	0.11	0.03	0.32	0.03	0.32
T_12_7	Antisocial	G_16_1	1.44E−03	0.02	0.47	0.16	0.27	0.16	0.47
T_12_7	Antisocial	G_28_15	3.32E−03	0.02	0.47	0.08	0.38	0.08	0.47
T_12_7	Antisocial	G_31_8	2.70E−05	0.02	0.47	0.09	0.54	0.09	0.54
T_12_7	Antisocial	G_37_14	3.41E−03	0.02	0.47	0.05	0.14	0.05	0.47
T_12_7	Antisocial	G_7_2	1.46E−03	0.02	0.47	0.09	0.23	0.09	0.47
T_12_7	Antisocial	G_7_3	5.81E−04	0.02	0.47	0.17	0.36	0.17	0.47
T_12_9	Antisocial	G_13_3	9.30E−05	0.00	0.24	0.03	0.32	0.03	0.32
T_12_9	Antisocial	G_26_14	2.35E−03	0.00	0.24	0.09	0.24	0.09	0.24
T_13_13	Reliable	G_5_3	3.68E−04	0.08	0.03	0.28	0.05	0.28	0.05
T_13_3	Antisocial	G_11_7	8.52E−04	0.02	0.55	0.08	0.38	0.08	0.55
T_13_3	Antisocial	G_13_3	1.51E−05	0.02	0.55	0.03	0.32	0.03	0.55
T_13_3	Antisocial	G_16_15	7.47E−05	0.02	0.55	0.16	0.15	0.16	0.55
T_13_3	Antisocial	G_21_16	4.37E−03	0.02	0.55	0.14	0.22	0.14	0.55
T_13_3	Antisocial	G_22_6	6.57E−04	0.02	0.55	0.08	0.16	0.08	0.55
T_13_3	Antisocial	G_25_3	1.04E−03	0.02	0.55	0.13	0.50	0.13	0.55
T_13_3	Antisocial	G_28_15	8.06E−07	0.02	0.55	0.08	0.38	0.08	0.55
T_13_3	Antisocial	G_31_8	2.52E−06	0.02	0.55	0.09	0.54	0.09	0.55
T_13_3	Antisocial	G_33_4	5.77E−04	0.02	0.55	0.00	0.33	0.02	0.55
T_13_3	Antisocial	G_35_22	1.49E−03	0.02	0.55	0.07	0.21	0.07	0.55
T_13_3	Antisocial	G_38_13	1.76E−03	0.02	0.55	0.13	0.20	0.13	0.55
T_13_3	Antisocial	G_7_2	1.04E−03	0.02	0.55	0.09	0.23	0.09	0.55
T_13_3	Antisocial	G_7_3	1.05E−04	0.02	0.55	0.17	0.36	0.17	0.55
T_13_3	Antisocial	G_9_2	1.70E−06	0.02	0.55	0.04	0.24	0.04	0.55
T_13_4	Sensitive	G_12_8	1.34E−04	0.28	0.05	0.09	0.03	0.28	0.05
T_13_4	Sensitive	G_25_3	3.27E−03	0.28	0.05	0.13	0.50	0.28	0.50
T_13_5	Reliable	G_38_17	1.01E−03	0.14	0.04	0.00	0.43	0.14	0.43
T_13_7	Sensitive	G_39_21	3.15E−04	0.24	0.05	0.11	0.07	0.24	0.07
T_13_9	Sensitive	G_31_8	1.36E−03	0.02	0.48	0.09	0.54	0.09	0.54
T_13_9	Sensitive	G_38_23	1.85E−03	0.02	0.48	0.05	0.49	0.05	0.49
T_13_9	Sensitive	G_7_2	2.67E−03	0.02	0.48	0.09	0.23	0.09	0.48
T_13_9	Sensitive	G_7_3	6.05E−06	0.02	0.48	0.17	0.36	0.17	0.48
T_14_1	Sensitive	G_12_11	4.06E−05	0.00	0.20	0.03	0.21	0.03	0.21
T_14_1	Sensitive	G_28_15	7.68E−04	0.00	0.20	0.08	0.38	0.08	0.38
T_14_1	Sensitive	G_7_2	5.15E−04	0.00	0.20	0.09	0.23	0.09	0.23
T_14_4	Reliable	G_21_18	6.57E−06	0.43	0.03	0.13	0.09	0.43	0.09
T_14_5	Antisocial	G_13_3	4.29E−06	0.00	0.41	0.03	0.32	0.03	0.41
T_14_5	Antisocial	G_31_8	2.62E−03	0.00	0.41	0.09	0.54	0.09	0.54
T_14_5	Antisocial	G_7_7	3.87E−03	0.00	0.41	0.03	0.10	0.03	0.41
T_14_5	Antisocial	G_9_2	1.36E−04	0.00	0.41	0.04	0.24	0.04	0.41
T_14_7	Antisocial	G_40_5	3.13E−03	0.02	0.36	0.06	0.38	0.06	0.38
T_14_8	Sensitive	G_30_28	1.21E−03	0.07	0.29	0.06	0.12	0.07	0.29
T_15_1	Antisocial	G_12_11	4.41E−03	0.13	0.35	0.03	0.21	0.13	0.35
T_15_1	Antisocial	G_21_16	5.43E−04	0.13	0.35	0.14	0.22	0.14	0.35
T_15_1	Antisocial	G_21_3	3.40E−03	0.13	0.35	0.10	0.23	0.13	0.35
T_15_1	Antisocial	G_33_4	1.93E−03	0.13	0.35	0.00	0.33	0.13	0.35
T_15_1	Antisocial	G_39_26	1.12E−03	0.13	0.35	0.20	0.30	0.20	0.35
T_15_12/T_11_2^i^	Sensitive	G_13_12	4.87E−04	0.02	0.39	0.23	0.31	0.23	0.39
T_15_12	Sensitive	G_13_12	4.05E−04	0.02	0.39	0.23	0.31	0.23	0.39
T_15_12/T_11_2^i^	Sensitive	G_7_3	4.74E−06	0.02	0.39	0.17	0.36	0.17	0.39
T_15_12	Sensitive	G_7_3	2.30E−05	0.02	0.39	0.17	0.36	0.17	0.39
T_15_13	Antisocial	G_12_8	3.64E−03	0.23	0.08	0.09	0.03	0.23	0.08
T_15_15	Reliable	G_12_8	3.10E−03	0.21	0.02	0.09	0.03	0.21	0.03
T_15_2	Reliable	G_21_18	2.81E−03	0.20	0.00	0.13	0.09	0.20	0.09
T_15_3	Antisocial	G_13_3	1.15E−03	0.06	0.05	0.03	0.32	0.06	0.32
T_15_4	Sensitive	G_13_3	1.60E−08	0.03	0.17	0.03	0.32	0.03	0.32
T_15_4	Sensitive	G_38_38	6.77E−04	0.03	0.17	0.00	0.16	0.03	0.17
T_15_4	Sensitive	G_7_2	4.07E−03	0.03	0.17	0.09	0.23	0.09	0.23
T_15_8	Sensitive	G_42_39	3.04E−03	0.35	0.06	0.16	0.11	0.35	0.11
T_3_1	Antisocial	G_35_7	2.11E−03	0.05	0.17	0.09	0.13	0.09	0.17
T_3_1	Antisocial	G_39_26	2.38E−03	0.05	0.17	0.20	0.30	0.20	0.30
T_3_3	Sensitive	G_13_3	6.18E−08	0.01	0.38	0.03	0.32	0.03	0.38
T_3_3	Sensitive	G_21_3	4.40E−03	0.01	0.38	0.10	0.23	0.10	0.38
T_3_3	Sensitive	G_28_15	1.77E−05	0.01	0.38	0.08	0.38	0.08	0.38
T_3_3/T_12_3^i^	Sensitive	G_31_8	1.78E−05	0.01	0.38	0.09	0.54	0.09	0.54
T_3_3	Sensitive	G_31_8	1.93E−05	0.01	0.38	0.09	0.54	0.09	0.54
T_3_3	Sensitive	G_38_38	7.60E−04	0.01	0.38	0.00	0.16	0.01	0.38
T_3_3	Sensitive	G_7_2	6.78E−04	0.01	0.38	0.09	0.23	0.09	0.38
T_3_3	Sensitive	G_9_2	3.80E−05	0.01	0.38	0.04	0.24	0.04	0.38
T_4_1	Reliable	G_12_8	3.15E−04	0.36	0.00	0.09	0.03	0.36	0.03
T_4_1	Reliable	G_8_8	2.45E−03	0.36	0.00	0.08	0.07	0.36	0.07
T_5_1	Reliable	G_20_2	3.95E−03	0.40	0.04	0.24	0.12	0.40	0.12
T_5_1/T_6_3^i^	Reliable	G_20_2	2.57E−03	0.40	0.04	0.24	0.12	0.40	0.12
T_6_1	Reliable	G_12_8	8.37E−08	0.22	0.00	0.09	0.03	0.22	0.03
T_6_1	Reliable	G_8_8	1.94E−04	0.22	0.00	0.08	0.07	0.22	0.07
T_11_7/T_4_3^i^	Sensitive	G_19_3	2.37E−04	0.04	0.47	0.08	0.17	0.08	0.47
T_11_7	Sensitive	G_22_6	7.90E−04	0.04	0.47	0.08	0.16	0.08	0.47
T_11_7/T_4_3^i^	Sensitive	G_33_4	1.66E−03	0.04	0.47	0.00	0.33	0.04	0.47
T_11_7/T_6_6^i^	Sensitive	G_33_4	1.47E−03	0.04	0.47	0.00	0.33	0.04	0.47
T_11_7/T_4_3^i^	Sensitive	G_35_22	4.76E−04	0.04	0.47	0.07	0.21	0.07	0.47
T_11_7/T_4_3^i^	Sensitive	G_39_26	4.12E−03	0.04	0.47	0.20	0.30	0.20	0.47
T_11_7/T_5_3^i^	Sensitive	G_39_26	1.34E−04	0.04	0.47	0.20	0.30	0.20	0.47
T_15_12	Sensitive	G_25_3	1.14E−03	0.02	0.39	0.13	0.50	0.13	0.50
T_15_12	Sensitive	G_38_23	1.65E−03	0.02	0.39	0.05	0.49	0.05	0.49
T_3_3	Sensitive	G_21_16	2.77E−03	0.01	0.38	0.14	0.22	0.14	0.38
T_3_3	Sensitive	G_41_33	1.88E−03	0.01	0.38	0.11	0.21	0.11	0.38
T_4_1	Reliable	G_5_3	1.62E−03	0.36	0.00	0.28	0.05	0.36	0.05
T_5_1	Reliable	G_12_1	2.17E−03	0.40	0.04	0.20	0.06	0.40	0.06
T_5_1	Reliable	G_13_12	2.96E−03	0.40	0.04	0.23	0.31	0.40	0.31
T_5_1/T_12_5^i^	Reliable	G_21_18	2.15E−04	0.40	0.04	0.13	0.09	0.40	0.09
T_5_1	Reliable	G_28_10	2.69E−03	0.40	0.04	0.15	0.15	0.40	0.15
T_6_1	Reliable	G_21_17	3.38E−03	0.22	0.00	0.18	0.09	0.22	0.09
T_6_1	Reliable	G_21_18	1.53E−03	0.22	0.00	0.13	0.09	0.22	0.09
T_6_4	Antisocial	G_14_12	2.60E−03	0.07	0.19	0.12	0.07	0.12	0.19
T_6_4	Antisocial	G_21_18	3.15E−03	0.07	0.19	0.13	0.09	0.13	0.19
T_6_4	Antisocial	G_36_18	1.52E−03	0.07	0.19	0.05	0.26	0.07	0.26
T_6_5	Antisocial	G_7_7	3.54E−04	0.02	0.36	0.03	0.10	0.03	0.36
T_6_5	Antisocial	G_13_3	6.37E−05	0.02	0.36	0.03	0.32	0.03	0.36
T_6_5	Antisocial	G_35_22	1.30E−03	0.02	0.36	0.07	0.21	0.07	0.36
T_7_3	Sensitive	G_7_3	7.96E−04	0.02	0.41	0.17	0.36	0.17	0.41
T_7_3	Sensitive	G_36_29	4.38E−03	0.02	0.41	0.08	0.48	0.08	0.48
T_7_3	Sensitive	G_38_23	3.87E−03	0.02	0.41	0.05	0.49	0.05	0.49
T_7_4	Sensitive	G_21_3	2.24E−03	0.00	0.26	0.10	0.23	0.10	0.26
T_8_6	Sensitive	G_7_2	7.69E−04	0.00	0.55	0.09	0.23	0.09	0.55
T_8_6	Sensitive	G_13_3	4.81E−07	0.00	0.55	0.03	0.32	0.03	0.55
T_8_6	Sensitive	G_31_8	5.66E−05	0.00	0.55	0.09	0.54	0.09	0.55
T_8_6	Sensitive	G_28_15	1.01E−03	0.00	0.55	0.08	0.38	0.08	0.55
T_8_6	Sensitive	G_7_3	4.98E−04	0.00	0.55	0.17	0.36	0.17	0.55
T_8_6	Sensitive	G_11_7	4.56E−04	0.00	0.55	0.08	0.38	0.08	0.55
T_8_6	Sensitive	G_21_16	2.97E−06	0.00	0.55	0.14	0.22	0.14	0.55
T_8_6	Sensitive	G_35_22	7.31E−04	0.00	0.55	0.07	0.21	0.07	0.55
T_8_6	Sensitive	G_38_38	3.66E−04	0.00	0.55	0.00	0.16	0.00	0.55
T_8_6	Sensitive	G_33_4	3.07E−04	0.00	0.55	0.00	0.33	0.00	0.55
T_8_6	Sensitive	G_25_3	6.21E−04	0.00	0.55	0.13	0.50	0.13	0.55
T_8_7	Sensitive	G_7_3	7.05E−04	0.00	0.46	0.17	0.36	0.17	0.46
T_8_7	Sensitive	G_41_33	3.85E−03	0.00	0.46	0.11	0.21	0.11	0.46
T_8_8	Reliable	G_13_3	2.97E−03	0.14	0.04	0.03	0.32	0.14	0.32
T_8_8	Reliable	G_25_20	5.20E−04	0.14	0.04	0.03	0.12	0.14	0.12
T_9_5	Reliable	G_28_10	3.59E−04	0.24	0.15	0.15	0.15	0.24	0.15
T_9_5	Reliable	G_33_33	5.10E−04	0.24	0.15	0.10	0.08	0.24	0.15
T_9_6	Antisocial	G_13_3	5.14E−09	0.00	0.65	0.03	0.32	0.03	0.65
T_9_6	Antisocial	G_31_8	5.76E−06	0.00	0.65	0.09	0.54	0.09	0.65
T_9_6	Antisocial	G_28_15	3.36E−05	0.00	0.65	0.08	0.38	0.08	0.65
T_9_6	Antisocial	G_7_3	1.89E−05	0.00	0.65	0.17	0.36	0.17	0.65
T_9_6	Antisocial	G_22_6	2.24E−03	0.00	0.65	0.08	0.16	0.08	0.65
T_9_6	Antisocial	G_11_7	1.30E−07	0.00	0.65	0.08	0.38	0.08	0.65
T_9_6	Antisocial	G_35_22	8.59E−06	0.00	0.65	0.07	0.21	0.07	0.65
T_9_6	Antisocial	G_33_4	2.90E−03	0.00	0.65	0.00	0.33	0.00	0.65
T_9_9	Sensitive	G_12_11	3.78E−03	0.05	0.10	0.03	0.21	0.05	0.21
T_9_9	Sensitive	G_13_3	8.87E−06	0.05	0.10	0.03	0.32	0.05	0.32
T_6_5	Antisocial	G_9_2	2.65E−04	0.02	0.36	0.04	0.24	0.04	0.36
T_8_6	Sensitive	G_9_2	2.30E−07	0.00	0.55	0.04	0.24	0.04	0.55
T_8_6	Sensitive	G_22_6	2.97E−06	0.00	0.55	0.08	0.16	0.08	0.55
T_9_6	Antisocial	G_9_2	2.50E−08	0.00	0.65	0.04	0.24	0.04	0.65
T_9_6	Antisocial	G_26_14	1.29E−04	0.00	0.65	0.09	0.24	0.09	0.65
T_8_1	Reliable	G_16_5	7.55E−04	0.26	0.00	0.09	0.15	0.26	0.15
T_8_2	Reliable	G_21_18	5.60E−05	0.30	0.02	0.13	0.09	0.30	0.09
T_11_7	Sensitive	G_41_37	8.75E−05	0.04	0.47	0.10	0.25	0.10	0.47
T_4_1	Reliable	G_37_6	4.52E−03	0.36	0.00	0.35	0.25	0.36	0.25

Association is measured by Fisher’s exact test (hypergeometric). Probabilities of well-being and ill-being are given for subjects in the character sets, the SNP sets, and subjects identified in both jointly. ^i^ indicates Temperament sets that are more specific than their parental sets, which are also selected

Clusters of individuals sharing SNPs and/or subjects (Fig. 1b) often had similar temperament profiles associated with particular molecular processes (Table 3, Supplementary Tables S4, S7). As predicted, each of the temperament profiles was strongly associated with regulation of synaptic plasticity and associative conditioning by genes regulating the Ras-MEK-ERK and PI3K-AKT-mTOR cascades in interaction with one another, Protein Kinases A, B (also known as AKT), and C, and various physiological and psychosocial stressors (Fig. 2c, Table 1, Supplementary Table S4).

Specific components of these complex molecular cascades distinguished each temperament profile (Supplementary Tables S4, S7). For example, SNP sets involving neuroexcitability (G_35_7, G_37_14), dopaminergic activation (G_16_1, G_35_22, G_39_26), and olfaction (G_7_7) were associated with the antisocial profile (Table 1, Fig. 1b, Supplementary Table S4). SNP sets involving resistance to aging and stress (G_12_8, G_20_2, G_21_17, G_30_10, G_33_33), cognitive flexibility (G_21_8, G_38_17), and cholinergic neuromodulation (G_13_10) were associated with the Reliable profile. SNP sets involving sensory sensitivity (G_38_21), susceptibility to fear conditioning (G_30_9, G_39_21), stress reactivity (G_7_2, G_11_7, G_26_14), and serotonin–cytokine interactions in response to stress (G_9_2) were associated with the Sensitive profile.

### Relations among SNP sets with one another and molecular processes

We found 17 single and disjoint nodes, and at least 3 sub-networks composed of highly connected nodes, shown in Fig. 1b (see Supplementary Information, 9. Identification of sub-networks). SNP sets G_8_8 (Inositol-Chemokine signaling), G_9_2 (Serotonin–Chemokine interaction), and G_7_3 (Neurogenesis) each represent the hub of sub-networks by their direct connections to 6 or 7 other SNP sets. These networks were relatively disjoint (i.e., sharing few SNPs and subjects; see Supplementary Section 6 (iv)), suggesting that these are distinct antecedents of personality.

### Heterogenic pathways influence the same temperament trait

The genes associated with each of the three temperament profiles were largely unique to that profile. 73.6% of the 736 genes associated with temperament were unique to a single temperament profile: 266 with reliable, 236 with sensitive, and 40 with antisocial (Supplementary Table S8). Consequently, there were multiple clusters of genes that lead to each individual temperament trait, as depicted in Fig. 1f. For example, high Novelty Seeking is a composite of individuals with the antisocial or sensitive temperament profiles because both are associated with features of high Novelty Seeking. Likewise, high Reward Dependence is a composite of individuals with Sensitive or Reliable profiles.

More generally, we refer to the multiple genotypic–phenotypic networks that contribute to individual traits as a pipeline, as depicted in Fig. 1f. The specific genes and molecular processes in the pipelines for each of the four temperament traits are described in Supplementary Tables S9–S11.

### Complex genotypic–phenotypic relationships influence health status

Combining genotypic and phenotypic information provided more information than either alone for both well-being (Fig. 1g vs. 1h) and ill-being (Supplementary Figures S3 vs. S4). When health status was based on the joint relationship of SNP sets and temperament sets, all three temperament profiles were well distinguished in terms of the probabilities of both ill-being (*p* < 1.58E−42, ANOVA statistics, Fig. 1c) and well-being (*p* < 1.05E−23, ANOVA, Fig. 1d). In contrast, when health status was based on temperament scores only, the probabilities of ill-being (*p*-value < 1.27E−06, ANOVA statistics, Supplementary Figure S5A) and well-being (*p*-value < 1.33E−05, ANOVA statistics, Supplementary Figure S5B) differentiated only the reliable profile from the other two.

We found 46 “switch” genes associated with temperament. These are a few genes in a particular SNP set whose presence or absence is associated with a switch in health status (Supplementary Table S12). These included 23 protein-coding genes, 10 lincRNAs, 4 other ncRNAs, 6 pseudogenes, 1 anti-sense, and 1 sense-intronic gene.

Overall about 67% of the 736 genes associated with temperament may be involved in regulatory processes: these included transcriptional regulators (10%), lncRNAs (14%), other RNA genes (5%), and targets of microRNAs (36%) as identified in the TRANSFAC® release 2017.1 database (Supplementary Table S13). We identified one microRNA (MIR7162) in association with temperament, and it targets 116 of the 736 genes we found associated with temperament in TRANSFAC.

### Replication of results in two independent samples

We tested the replicability of our findings in the Finnish study by carrying out the same analyses in the German and Korean samples. All but one (98%) of the 51 SNP sets associated with temperament in the Finnish sample were identified in one or both of the replication samples: 40 were identified in both the Korean and German samples, 5 in the Korean sample only, and 5 in the German sample only (Supplementary Table S14). We also found that all but one (98%) of the 44 Temperament Sets associated with SNP sets in the Finnish sample were replicated in the other samples: 31 in both, 7 in Korean sample only, and 5 in the German sample only (Table S15).

Overall, the genotypic–phenotypic relations between the SNP and temperament sets identified in the Finnish sample were closely matched by those observed in both the Korean study (89%) and in the German (76%) study (Supplementary Table S16). The genotypic–phenotypic relations of people with reliable and sensitive temperaments were strongly replicated in both samples. However, at least two antisocial temperament sets strongly associated with ill-being and with several SNP sets were missing in the German sample, which had been screened to exclude anyone with a history of psychiatric illness in themselves or their first-degree relatives. The absence of these unhealthy temperament sets reduced replicability of genotypic–phenotypic relations in the German sample as expected (Supplementary Figures S6). The strength of the identity of replicated sets was calculated using Hypergeometric statistics and Multi-objective optimization techniques (see Pareto values in Supplementary Tables S17, S18).

Prior literature reporting associations with TCI-related key words were systematically surveyed from PubMed to identify genes that had been reported to be associated with TCI traits (Supplementary Tables S19, S20). We found that 120 of our detected genes were related to genes, family of proteins, or pathways of genes previously associated with TCI traits (Supplementary Table S19). Among the genes in temperament-related SNP sets, we also detected 74% of the 111 genes that had been previously associated with TCI temperament or character traits, and 78% of the 74 genes that had previously been reported in association with TCI temperament traits (Supplementary Table S20). Considering all 111 genes previously associated with any TCI traits in a multi-omic approach (Supplementary Table S20), we recovered 6 genes exactly, another 32 variants from the same family of proteins, and another 44 genes in the same molecular pathway previously reported.

### Estimation of heritability and environmental influences

The heritability of temperament controlling for outliers was estimated as 48% in the Finnish sample, 53% in the German sample, and 37% in the Korean sample (Supplementary Table S21). In addition, 87% of the SNP sets were strongly associated with the empirical temperament index (5E−08 > *p*-value > 5E−73). In other words, the SNPs that comprise the different SNP sets strongly distinguished the temperament features of the subjects in each set, indicating that each individual SNP set contributed significantly to explain the total distributed heritability (Supplementary Section 9). Consequently, when the genotypic sets were used to classify the well- and ill-being of the subjects using the PGMRA classifier, the predicted values were highly accurate (average Areas Under Curve of the classifications were 0.940 and 0.922, respectively) (Supplementary Figure S8).

We also considered environmental influences in the Finnish sample. There were direct associations of sets of environmental influences in childhood and adulthood (Supplementary Table S22A) with temperament sets (Supplementary Table S22B) and with SNP sets (Supplementary Table S22C). The impact of these correlations was small, so the heritability estimate was still 46–52% in the Finnish sample when adjusted for gene–environment correlation (Supplementary Table S21).

Furthermore, 12 novel associations between SNP sets and temperament sets were uncovered when environmental influences were used as mediators (Supplementary Table S22D). Seven SNP sets associated with the antisocial profile depended on exposure to low parental income during childhood, stressful life events in adulthood, and rural residence in childhood or adulthood (*p* < 3.4E−03 to 6.3E−04). Two SNP sets associated with sensitive profiles depended on the experience of tolerance and low income in childhood (*p* < 9.7E−04 to 4.7E−05). One SNP set associated with reliable profiles depended on high parental income throughout childhood (*p* < 1.5E−04).

## Discussion

SNPs that map to 736 genes explained 48% of the variability in temperament in the Finnish sample, thereby accounting for nearly all the heritability of human temperament expected from twin studies. More specifically, most of the genes that we identified in a strictly data-driven manner are known to regulate synaptic plasticity, associative conditioning, and related processes of stress reactivity and neurotransmission. These findings confirm our hypothesis that the highly conserved molecular processes that regulate associative conditioning in experimental animals account substantially for the heritability of human temperament. Our findings are supported in independent replications by GWAS and by independent studies of gene expression during habit learning in experimental animals [7, 32, 33].

### Molecular pathways for temperament and associative conditioning

Most of the SNP sets associated with temperament were involved in the regulation of habit learning and synaptic plasticity in response to extracellular stimuli mediated mainly by the Ras-MEK-ERK and the PI3K-AKT-mTOR cascades (Table 1, Fig. 2c). As predicted, these main pathways of fast adaptive response operated in conjunction with related processes for stress reactivity, neurotransmission, chromatin plasticity, neuronal and glial growth, myelination, neuroprotection, and energy production (Table 1, Supplementary Tables S4–S6). The identified pathways for associative conditioning are known to intersect to regulate each other and to co-regulate downstream functions [52], as illustrated specifically in Fig. 2c. The mechanisms for integration of the ERK and PI3K cascades include mechanisms for cross-activation, cross-inhibition, negative feedback, and positive and negative influences that converge on the same complex (e.g., mTOR in Fig. 2c). In addition, protein kinases A, B (also known as AKT), and C that regulate these pathways are rather non-selective [52]. Such interactions are expected to produce complex genotypic–phenotypic relationships, as we observed.

These findings about specific molecular pathways for human temperament have important implications. First, they confirm our hypothesis that the human temperament is based on the highly conserved mechanisms for habit learning. This supports a precise definition of temperament in terms of associative conditioning [17, 18]. Second, the independent experimental support for specific molecular pathways for associative conditioning provides support for the validity of the strictly data-driven method we used to analyze and interpret genome-wide association data.

These results should encourage widespread use of PGMRA for analysis of complex phenotypes in a variety of settings, including GWAS [54, 55] and neuroimaging [53]. For example, PGMRA provides an effective way to allow for epistasis and gene–environment interactions that are prominent in complex phenotypes, thereby overcoming the hidden heritability problem (that is, the consistent inability to account for most of the heritability of complex traits when only the average effects of genes are considered). The generalized clustering method implemented in PGMRA can be interpreted as a deep unsupervised NMF learning process that can identify clusters of individuals with distinct features from various types of information, such as the genotypes, phenotypes, and environments (Supplementary Figure S1). Such clusters, SNP sets, and temperament sets can be used as auto-encoders used by recommender systems in precision medicine [55].

### Strengths and limitations

The major strength of these findings is the strong replicability of the findings in three independent samples from different cultures and in independent studies of gene expression during behavioral conditioning of experimental animals. While it is true that cluster analysis is a hypothesis-generating method in which there is no unique solution to the number of clusters, which features are relevant for a cluster, or the degree of homogeneity to be demanded for each cluster, PGMRA included a practical and robust solution for each of these problems [53, 54].

### Conclusions and recommendations for future research

We were able to describe and replicate the complex genotypic–phenotypic risk architecture of temperament in three independent samples of people. Our unbiased data-driven findings confirm the hypothesis that temperament is based on associative conditioning and related processes, particularly stress reactivity in response to extracellular stimuli. We have found that different molecular and cognitive processes are associated with character [40], but health status depends on genotypic–phenotypic relations that influence both temperament and character. Therefore, we recommend further work to examine the overlap and interactions between temperament and character.

## Electronic supplementary material

Supplementary Information

Supplementary Figure S1A

Supplementary Figure S1BC

Supplementary Figure S1D-F

Supplementary Figure S2

Supplementary Figure S3

Supplementary Figure S4

Supplementary Figure S5

Supplementary Figure S6

Supplementary Figure S7

Supplementary Figure S8

Supplementary Figure S9

Supplementary Table S1

Supplementary Table S2

Supplementary Table S3

Supplementary Table S4

Supplementary Table S5

Supplementary Table S6

Supplementary Table S7

Supplementary Table S8

Supplementary Table S9

Supplementary Table S10

Supplementary Table S11

Supplementary Table S12

Supplementary Table S13

Supplementary Table S14

Supplementary Table S15

Supplementary Table S16

Supplementary Table S17

Supplementary Figure S18

Supplementary Table S19

Supplementary Table 20

Supplementary Table S21

Supplementary Table S22
