# Supplementary figures and images for: Uncovering the complex genetics of human temperament
Source: Mol Psychiatry. 2018 Oct 2;25(10):2275–94. doi: 10.1038/s41380-018-0264-5 (PMC7515831; doi:10.1038/s41380-018-0264-5)

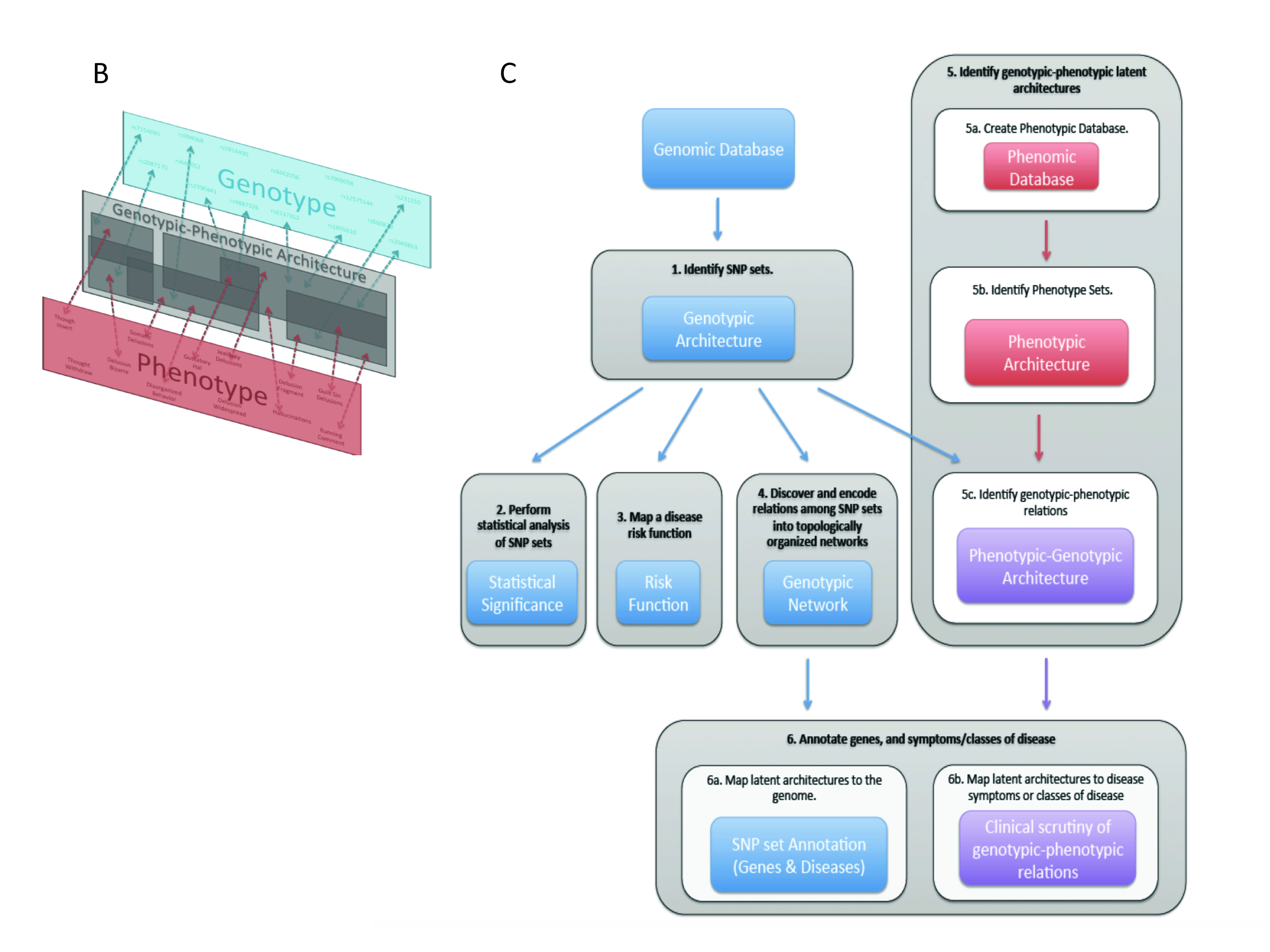

Supplement: Supplementary file 3 — Supplementary Figure S1BC [file 41380_2018_264_MOESM3_ESM.tif]

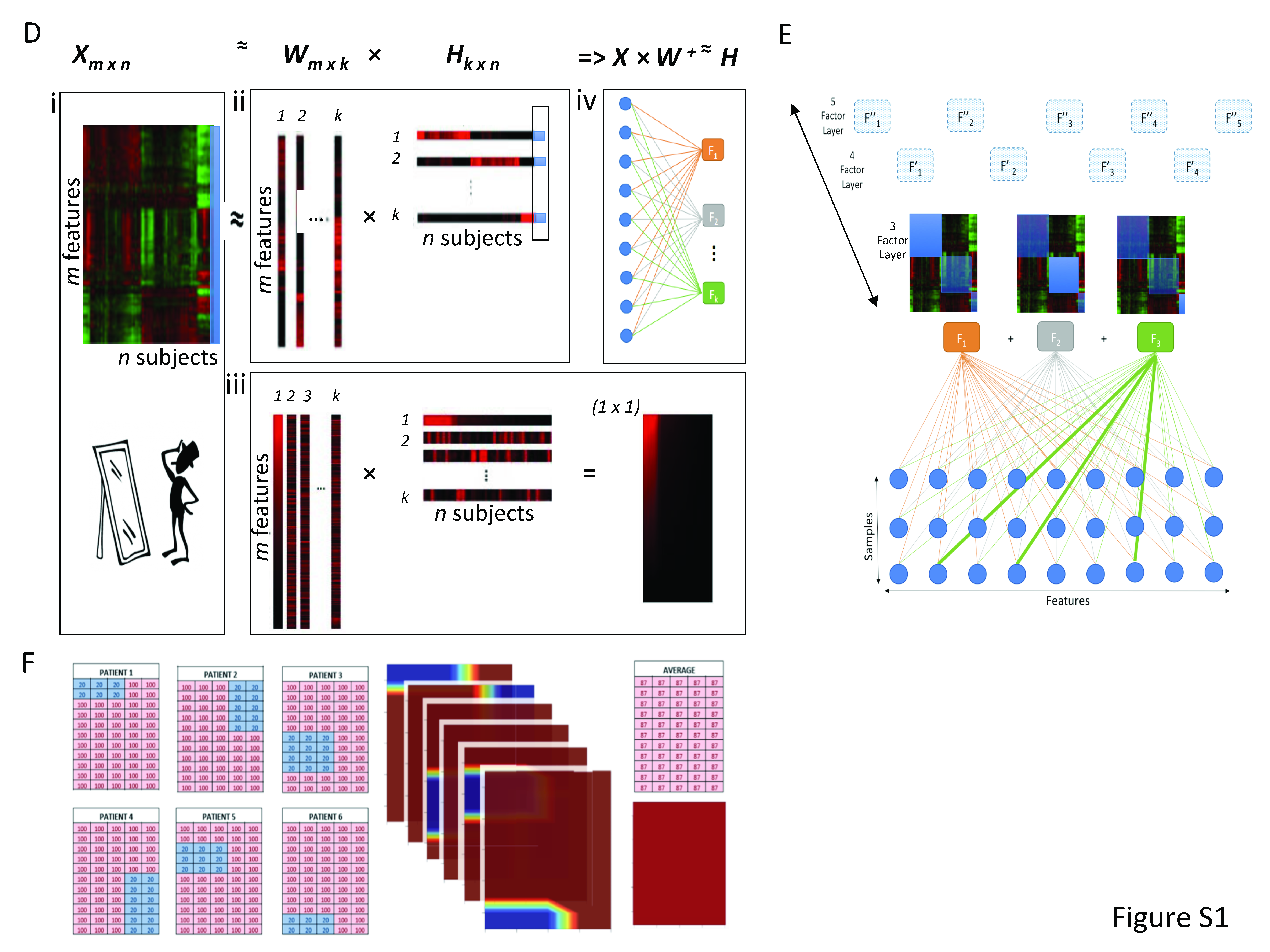

Supplement: Supplementary file 4 — Supplementary Figure S1D-F [file 41380_2018_264_MOESM4_ESM.tif]

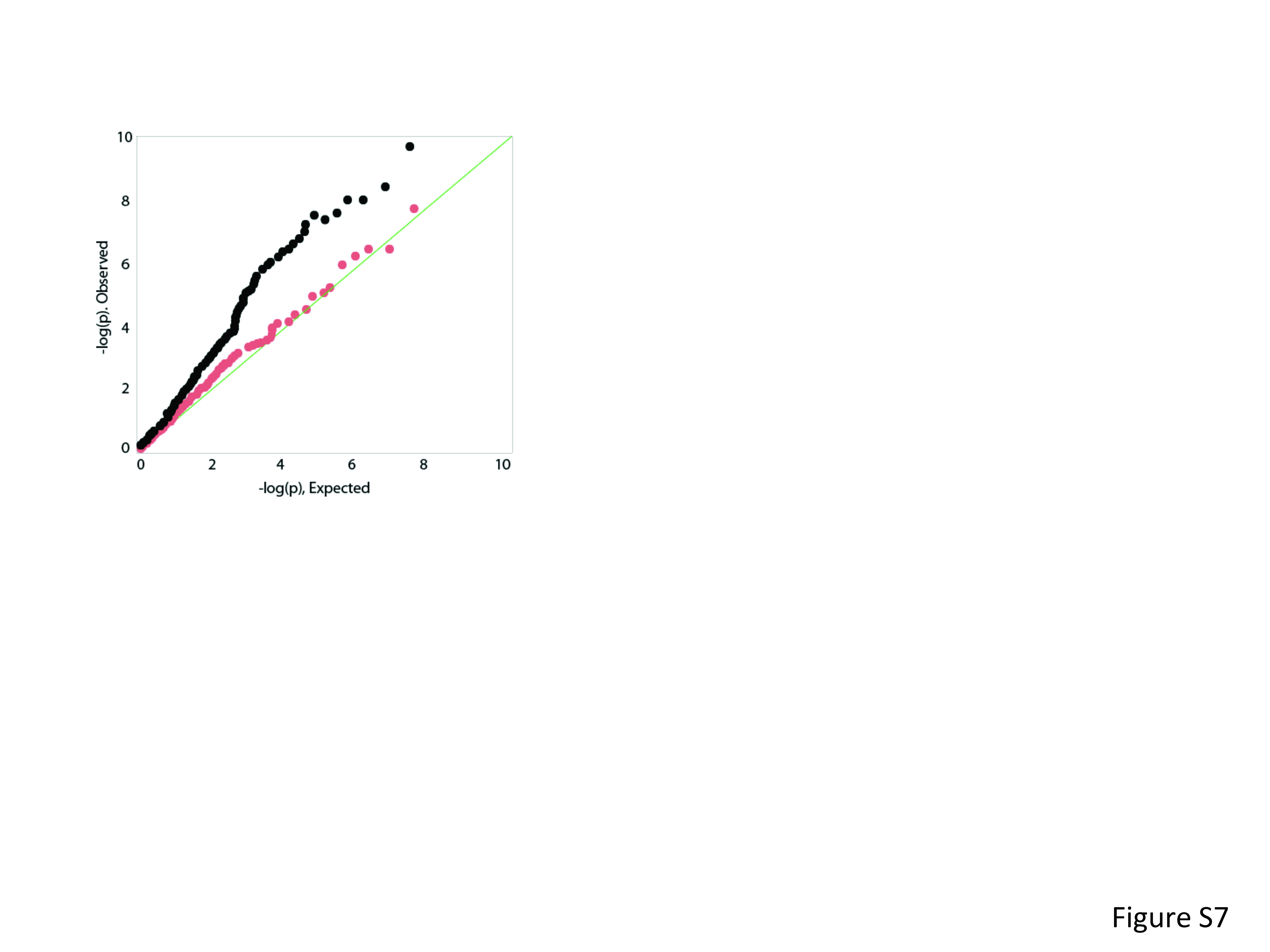

Supplement: Supplementary file 10 — Supplementary Figure S7 [file 41380_2018_264_MOESM10_ESM.tif]

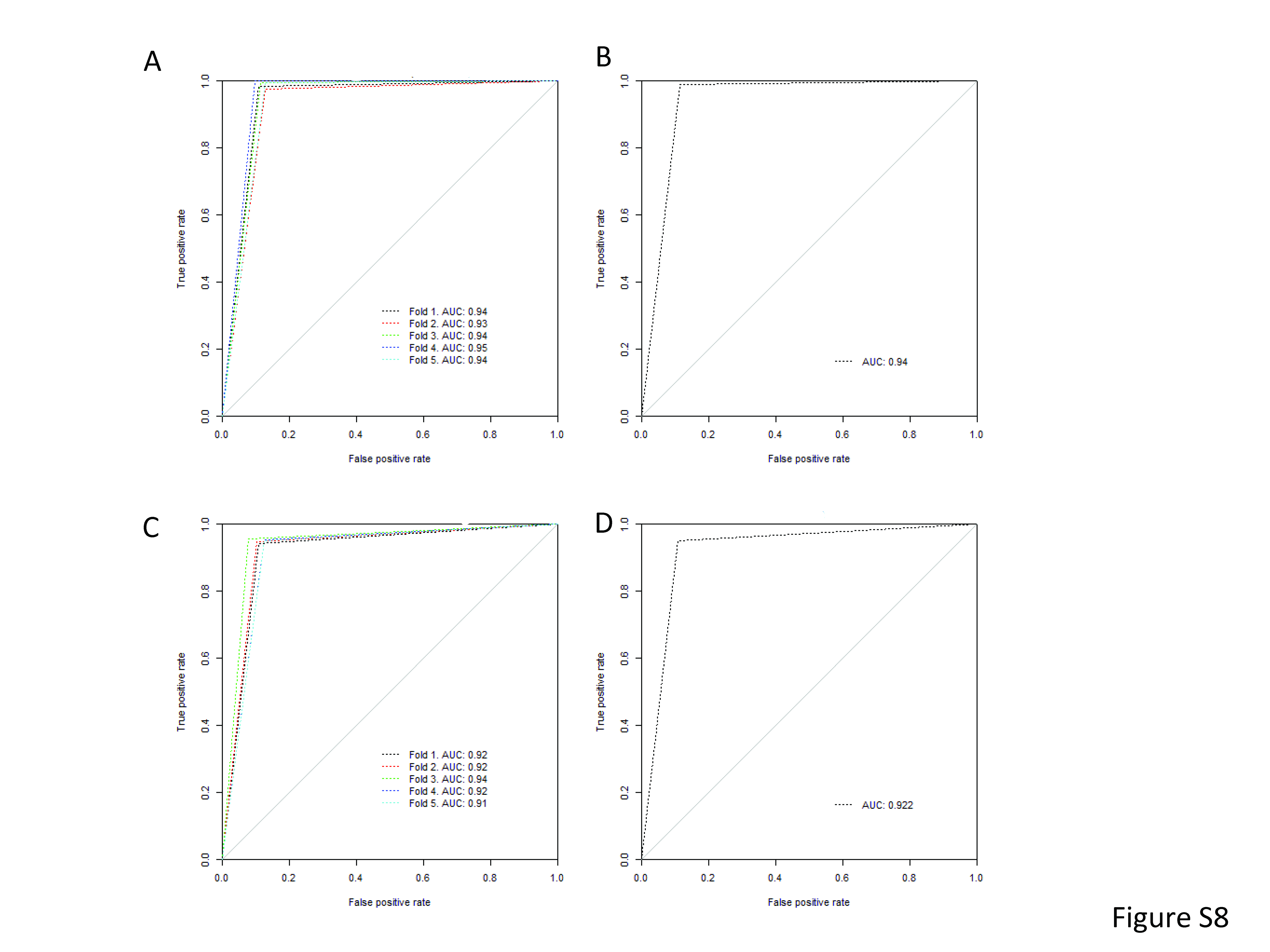

Supplement: Supplementary file 11 — Supplementary Figure S8 [file 41380_2018_264_MOESM11_ESM.tif]
